# Strategically examining the full-genome of dengue virus type 3 in clinical isolates reveals its mutation spectra

**DOI:** 10.1186/1743-422X-2-72

**Published:** 2005-08-24

**Authors:** Day-Yu Chao, Chwan-Chuen King, Wei-Kung Wang, Wei-June Chen, Hui-Lin Wu, Gwong-Jen J Chang

**Affiliations:** 1 Institute of Epidemiology, College of Public Health, National Taiwan University (NTU), Taipei, Taiwan (100), Republic of China (R.O.C.); 2 Institute of Microbiology, College of Medicine, NTU, Taipei, Taiwan (100), Republic of China (R.O.C.); 3 Dept. of Parasitology, Chang Gung College of Medicine and Technology, Kwei-San, Tao-Yuan, Taiwan (100), Republic of China (R.O.C.); 4 Hepatitis Research Center, NTU Hospital, Taipei, Taiwan (100), Republic of China (R.O.C.); 5 Division of Vector-Borne Infectious Diseases, National Center for Infectious Diseases, Centers for Disease Control and Prevention (CDC), Fort Collins, USA

**Keywords:** Quasispecies, mutation spectra, micro-evolution of dengue virus serotype 3, dengue hemorrhagic fever (DHF), sequence diversity, Taiwan

## Abstract

**Background:**

Previous studies presented the quasispecies spectrum of the envelope region of dengue virus type 3 (DENV-3) from either clinical specimens or field-caught mosquitoes. However, the extent of sequence variation among full genomic sequences of DENV within infected individuals remains largely unknown.

**Results:**

Instead of arbitrarily choosing one genomic region in this study, the full genomic consensus sequences of six DENV-3 isolates were used to locate four genomic regions that had a higher potential of sequence heterogeneity at capsid-premembrane (C-prM), envelope (E), nonstructural protein 3 (NS3), and NS5. The extentof sequence heterogeneity revealed by clonal sequencing was genomic region-dependent, whereas the NS3 and NS5 had lower sequence heterogeneity than C-prM and E. Interestingly, the Phylogenetic Analysis by Maximum Likelihood program (PAML) analysis supported that the domain III of E region, the most heterogeneous region analyzed, was under the influence of positive selection.

**Conclusion:**

This study confirmed previous reports that the most heterogeneous region of the dengue viral genome resided at the envelope region, of which the domain III was under positive selection pressure. Further studies will need to address the influence of these mutations on the overall fitness in different hosts (i.e., mosquito and human) during dengue viral transmission.

## Background

Dengue viruses (DENV), which consisted of four antigenically distinct serotypes (DENV-1, 2, 3 and 4), are the most important arthropod-borne viruses affecting humans. After infection, it may result in dengue fever (DF), dengue haemorrhagic fever (DHF), dengue shock syndrome (DSS) or death [1,2]. It is estimated that close to 50–100 million cases of DF and 30,000 fatal cases of DHF/DSS occur annually in tropical and subtropical regions. With the increased numbers of dengue patients, it is indicated the global expansion of epidemic areas, and increased frequencies of severe DHF/DSS and case fatality [3]. Considerable efforts have been devoted to developing vaccines to prevent dengue, but the success of the vaccines will be dependent on the vaccine strain chosen to direct against the diversity and evolution of DENV genome.

DENV belongs to the genus *Flavivirus*, family *Flaviviridae*, possessing a positive-sense, single-stranded RNA genome, which is approximately 10,700 bases in length and contains a single open reading frame [4]. A single polyprotein translated from the viral RNA is cleaved into 3 structural proteins [capsid (C), premembrane (prM) and envelope (E) protein] and 7 nonstructural proteins (NS), with the gene order as 5'-C-prM/M-E-NS1-NS2A-NS2B-NS3-NS4A-NS4B-NS5-3'. Like many RNA viruses, the genomic sequence of a single DENV isolate exists in nature as a collection of highly similar but not identical variants known as quasispecies due to its high average mutation rate of 10^-3 ^to 10^-5^substitution per nucleotide copied and per round of replication [5,6]. Previous studies using a clonal sequencing approach amplified viral RNA directly from DENV-3 infected patients' plasma and the extent of sequence heterogeneity in the envelope region with mean pairwise difference ranging from 0.21 to 1.67% have been observed [7]. There are obvious reasons for selecting the E gene region for this study, mainly due to its important biological functions such as receptor-mediated endocytosis, virus-induced cellular tropism and eliciting neutralization antibodies. However, one cannot exclude the biological significance of the sequence heterogeneity in other genomic regions including non-structural (NS) proteins, 5' and/or 3' non-coding regions (NCR). The well-studied example of hepatitis C virus (HCV) demonstrated that the quasispecies dynamics and composition of the NS5A region may play a role in disease prognosis and in response to interferon and ribavirin therapy [8]. Although the previous attempt to correlate the sequence heterogeneity of the capsid gene with NS protein 2B gene region of DENV-3 has observed very similar sequence heterogeneity with mean pairwise p-distance 0.12–1.2% [9], the extent of sequence variation among full genomic sequences of DENV within infected individuals remains largely unknown. Thus, it is important to address whether the evidence of different evolutionary processes, such as adaptive evolution, shape the population genetics of DENV at specific genomic regions other than the E region.

An outbreak of DHF, attributed to genotype 2 of DENV-3, resulted in 111 DF and 23 DHF cases in Tainan (southern Taiwan) from October 1998 to January of 1999 [10]. DENV-3 was the only serotype isolated during this outbreak, and the seroepidemological study clearly demonstrated that DHF cases were not associated with secondary DENV infection [10]. Here we report the selection of the most prominent variable regions identified by the full-genomic sequencing of DENV isolates from six clinical patients during this outbreak. The application of the clonal sequencing of those variable regions enabled us to study quasispecies structure of DENV isolates and to provide a better understanding of the changes in mutation spectrum at the clonal level and virus evolution.

## Results

### Heterogeneous regions identified at full genomic scale of DENV-3

**Figure 1:**
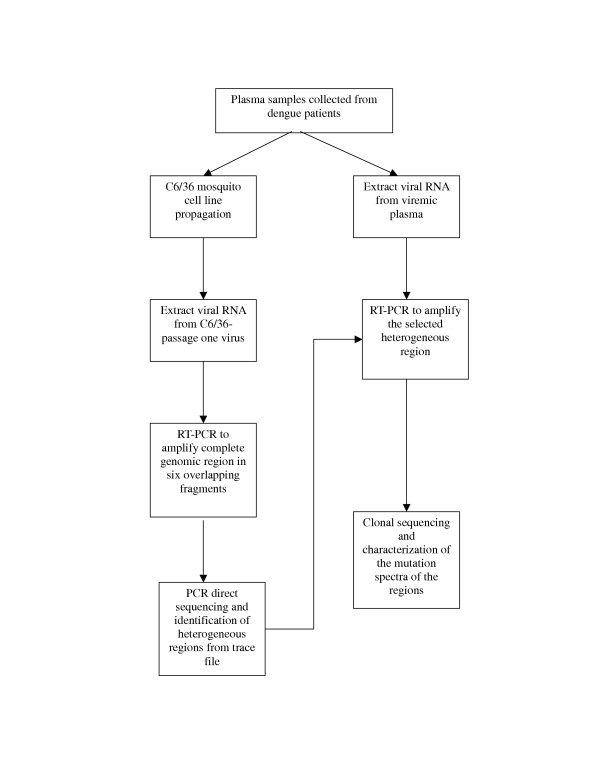
Strategy in clonal-sequencing the whole genome of genomic RNA of DENV-3.

To identify the potential heterogeneous regions of DENV-3 in the whole-genomic scale, acute-phase plasma samples were obtained from six dengue patients, including three DF (designated 1F, 2F and 3F) and three DHF patients (designated 1H, 2H and 3H). The sequencing strategy is depicted in Fig 1. These patients' plasma samples were used to infect the C6/36 mosquito cell line to obtain sufficient viral genomic RNA for full-genomic consensus sequencing for the identification of regions with sequence heterogeneity for follow-up clonal sequencing.

The consensus sequence similarity of these six viruses was as high as 99.73%. The 2H and 3H virus each had two silent changes at nucleotide positions of 808 (G to A), 9979 (T to C), 4204 (C to T) and 8785 (T to C), respectively (Table 1). There were no consistent nucleotide changes that might correlate with disease severity among paired viruses using this consensus sequencing approach. However, the potential heterogeneous sequence regions were clearly observed and identified by close examination of the overlapping chromatogram files using the SeqMan program in the Lasergene software package (DNASTAR inc., Madison, WI). Special attention was paid to identify the regions which consistently presented mixed-chromatographic peaks in the respective trace files obtained from at least two independent sequencing primers. These potential heterogeneous regions, located at C/PrM, E, NS3 and NS5 genes (Table 1), were selected for the clonal sequencing analysis. Five genomic fragments were amplified directly from six patients' viremic plasma by five flanking primer pairs (Table 4) at nucleotide position of 1–764 (5'NCR/C/prM), 1259–2550 (E/NS1), 5443–6337 (NS3) and 8501–10316 (NS5/3'NCR) using Titan™ one tube RT-PCR System (Boehringer Mannheim). After excluding the primer sequences, the C/PrM region was 752 nucleotides in length with 225 amino acids in the coding region; the E/NS1 region was 1239 nucleotides in length covering 413 amino acids which included 40 amino acids at the N terminal end of NS1 protein, the NS3 region was 866 nucleotides in length covering 288 amino acids, and the NS5 region was 1791 nucleotides in length with 586 amino acids in viral coding sequences.

**Table 1:** Identification of the positions of potential heterogeneity nucleotide sequence by the full genome consensus sequence of DENV-3^a ^viruses isolated during 1998–1999 dengue outbreak in Taiwan.

**Virus ID**	**Disease Status**^c^	**Nucleotide Changes at Positions Indicated**^b^													
		
		320–322	444–445	808	1693	1716	4204	5322	6045	6079	8785	9076	9979	10105	10128
**1F**	**DF**	RRR	TG (CA)	G	G(C)	C(T)	C	T	A	C	T	T	T	C	C
**1H**	**DHF**	GGG	TG	G	C	C(T)	C	T	A	C	T	T	T	C	C
**2H**	**DHF**	GGG	TG	A	G	C(T)	C	T	A	C	T	T(A)	C	T	C
**2F**	**DF**	GGG	TG	G	G	C(T)	C	T	A	C	T	T	T	C	C
**3H**	**DHF**	GGG	TG	G	G(C)	C(T)	T	T(C)	A(C)	C(T)	C	T	T	C	C
**3F**	**DF**	GGG	TG	G	G	C(T)	C	T	A	C	T	T	T	C	C(T)

^a^Nucleotide in parentheses indicated "mix nucleotide sequence" based on mix chromatographic signals in the sequencing trace file and nucleotide position number referred to the reference strain H87 of DENV-3 (genebank accession number: M93130)

^b ^DENV-3 viruses were isolated from the plasma of six dengue patients by one passage in the C6/36 mosquito cell culture.

^c ^Disease status was classified based on WHO criteria [30]. DF: dengue fever; DHF: dengue hemorrhagic fever.

**Table 4:** The Oligonucleotide primers and conditions used for RT-PCR of full-length genome of DENV-3

**PCR Primer**^a^	**Sequence (5' → 3')**	**Genome Position**^b^	**Size(nt)**^c^
P1A	AGT TGT TAG TCT RCG TGG	1–18	1181
CP1181B	TCC ARG CAC CTT CAG ATG	1181–1199	
DC530A	AAC AWR TGC ACC CTC	540–555	1164
CDC1694B	TGC ATK GCT CCT TCT TGR	1694–1712	
P1259A	GGC AAG GGA AGC TTG GTG ACA TGC GC	1259–1285	1244
CDC2503B	GGG AGT CTG CTT GGA ATT	2503–2521	
DC2171A	GCC ATT CTR GGW GAC ACC GCY TGG GA	2171–2197	1246
CDC3417B	TCT CTT CTT TGT CMT TCA	3417–3435	
D3-3142A	CCA AAG AGT CTA GCT GGT CC	3142–3162	1535
D3-4677B	CAT TGT GCG TCA ACA CTG CC	4677–4697	
d3NS2B1A	AGC TGG CCA CTG AAT GAG G	4124–4143	1562
D35686B	CAA AAG TCT TCC TAC TAA GTT G	5686–5708	
D3-5443A	GCC GCA ATT TTC ATG ACA	5443–5461	2034
D3-7477B	AAC AGC TAT CGT GGT GTT CC	7477–7497	
d37246A	AAG AAT CCA ACG GTG GAT GG	7246–7266	1454
d38750B	TCC CTT GTG CAT AAT CTG GG	8750–8770	
d38501A	CAG GCT CAG CCT CCT CC	8501–8518	1654
d310316B	GCT TCT TCC GTA CTG TGG C	10316–10335	
d39991A	CTT ACT GTC TGG AAC AGG G	9991–10010	648
d310688B	GTT GAT TCA ACA GCA CCA TTC	10688–10709	

a Primer names with A in the end indicate a viral-sense orientation; names with B in the end indicate a complementary sense orientation

b Genome positions are given according to the published sequence of strain H87 of dengue virus serotype 3

c nt indicated nucleotide

### Clonal sequencing of the heterogeneous regions among dengue viral genomes

The pCRII-TOPO™ T/A cloning kit (Invitrogen, San Diego, CA) was used to clone PCR products representing heterogeneous sequence regions identified by the consensus sequencing as described previously [7]. At least 20 to 30 clones containing the PCR amplicons from four heterogeneous gene regions (C-PrM, E, NS3 and NS5) were sequenced, aligned, and analyzed using the program GCG and MEGA v3.0 [11]. In general, the transitional substitutions were higher than transversional substitutions for all samples analyzed. The transitional changes (A to G or T to C) constituted overall substitution rate of 72.8 ± 5.1%, 73.7 ± 11% at C/PrM, E of structural proteins, and the NS3 and NS5 regions had relatively lower such changes of 63.2 ± 7.5%, 44.7 ± 18%. The lowest nucleotide mutation frequencies were observed in NS3 region with a mean ± standard deviation (SD) of 0.6 ± 0.3 × 10^-3 ^for all clones analyzed, followed by C/PrM (1.2 ± 0.15 × 10^-3^), NS5 (1.5 ± 0.4 × 10^-3^) and envelope region (1.8 ± 0.8 × 10^-3^), which was statistically significant (p < 0.01). Similarly, the substitution frequencies of amino acids were also variable among the viral genome regions with the lowest frequency observed in NS3 (1.3 ± 0.4 × 10^-3^), followed by C/PrM (2.3 ± 0.3 × 10^-3^), E (3.1 ± 1.8 × 10^-3^) and NS5 region (3.3 ± 0.8 × 10^-3^) (Table 2).

**Table 2:** Sequence diversity (mean p-distance) among different genomic regions of DENV-3

			**Nucleotide**					**Amino acid**			
				
Virus ID No.	Region	No of Clones	No of change/total	Mutation frequency^a ^(10^-3^)	A→G or U→C^b ^(%)	Mean p-distance^c^(10^-3^)	Range (10^-3^)	No of change/total	Mutation frequency^a^(10^-3^)	Mean p-distance^c^(10^-3^)	Range (10^-3^)
1F	C/PrM	23	24/17733	1.4	66.7	2.67	0–6.7	13/5175	2.5	5	0–8.9
1H	C/PrM	29	29/22359	1.3	69	2.45	0–6.7	17/6525	2.6	5.12	0–22.2
2H	C/PrM	21	16/16191	1.0	81.3	2.03	0–6.7	9/4725	1.9	3.81	0–13.3
2F	C/PrM	22	18/16962	1.1	72.2	2.17	0–6.7	10/4950	2.0	4	0–13.3
3H	C/PrM	13	11/10023	1.1	72.7	1.81	0–4	7/2925	2.4	4.7	0–17.8
3F	C/PrM	24	24/18504	1.3	75	2.55	0–8	13/5400	2.4	4.8	0–17.8
Mean ± std	C/PrM	22		1.2 ± 0.15	72.8 ± 5.1	2.28 ± 0.3			2.3 ± 0.3	4.6 ± 0.5	
											
1F	E/NS1	26	40/32240	1.2	72.5	2.23	0–6.5	21/10192	2.1	4.3	0–10.2
1H	E/NS1	13	47/16250	2.9	61.7	3.82	0–6.4	17/5096	3.3	6.6	0–7.6
2H	E/NS1	25	33/16250	2.0	93.9	3.88	0–5.6	34/9800	3.5	6.4	0–12.6
2F	E/NS1	13	15/22103	0.7	66.7	3.18	0–2.8	4/5096	0.8	7.6	0–9
3H	E/NS1	23	44/20240	2.2	72.7	3.83	0–4.8	55/9016	6.1	6.2	0–12.6
3F	E/NS1	20	39/24960	1.6	74.4	2.56	0–6.8	20/7840	2.6	4.8	0–21.7
Mean ± std	E/NS1	20		1.8 ± 0.8	73.7 ± 11	3.7 ± 0.7			3.1 ± 1.8	6 ± 1.2	
1F	NS3	19	5/17005	0.3	60	0.9	0–3.5	8/4978	1.6	3.3	0–11.4
1H	NS3	27	16/24165	0.7	56.3	1.2	0–4.6	11/7074	1.6	3.1	0–11.4
2H	NS3	26	9/23270	0.4	33.3	1.2	0–4.6	6/6812	0.9	3.2	0–11.5
2F	NS3	25	16/22375	0.7	40	2.4	0–5.8	12/6550	1.8	5.7	0–15.3
3H	NS3	23	25/20585	1.2	16	1.6	0–4.6	6/6026	1.0	2.0	0–11.4
3F	NS3	18	8/16110	0.5	62.5	1.0	0–4.6	4/4716	0.8	1.6	0–11.4
Mean ± std		23		0.6 ± 0.3	44.7 ± 18	1.4 ± 0.6			1.3 ± 0.4	3 ± 1.4	
											
1F	NS5	13	29/23335	1.2	69	1.7	0–4.5	17/7748	2.2	4.1	0–8.4
1H	NS5	17	32/30515	1.0	71.9	2.4	0–5.6	31/10132	3.1	3.5	0–8.4
2H	NS5	18	51/32310	1.6	64.7	3.0	0–8.9	33/10728	3.1	6.1	0–18.5
2F	NS5	25	60/44875	1.3	65	2.5	0–5.6	47/14900	3.2	3.6	0–8.4
3H	NS5	16	64/28720	2.2	56.3	4.3	0–7.3	46/9536	4.8	6.8	0–15.2
3F	NS5	26	69/46670	1.5	52.2	3.7	0–6.7	52/15496	3.4	6.5	0–11.8
Mean ± std		19		1.5 ± 0.4	63.2 ± 7.5	3.1 ± 0.9			3.3 ± 0.8	5.1 ± 1.5	

^a^Mutation frequency is defined as the proportion of mutations relative to the consensus nucleotide or amino acid sequence for each patient and calculated by dividing the number of mutations relative to consensus by the total number of nucleotides or amino acid sequenced in each sample.

^b ^Percentage of A→G or U→C transitional mutations

^c ^p-distance is calculated by pairwise comparison of nucleotide or amino acid sequences between clones by the program MEGA d Indicated the virus derived from mosquito inoculation by C6/36-passaged one virus of patient ID#1H.

The mean pairwise p-distance as described in the previous study [7] was employed to compare the extent of sequence variation among different viral genome regions. Consistently, NS3 had the lowest pairwise p-distance among NS5, C/PrM or E protein. The average mean p-distance in nucleotides and SD for NS3, C-PrM, NS5, and E were 1.4 ± 0.6 × 10^-3^, 2.3 ± 0.3 × 10^-3^, 3.1 ± 0.9 × 10^-3^, and 3.7 ± 0.7 × 10^-3^, respectively. At the amino acid level, NS3 also had the lowest mean p-distance (3 ± 1.4 × 10^-3^) and E proteins had the highest variability (6 ± 1.2 × 10^-3^) (Table 2). The difference of mean p-distance in nucleotides or amino acids among different genes was statistically significant (p < 0.01). No consistent correlation between any two different genes from the same human isolates with the extent of the nucleotide heterogeneity could be made. This would suggest that different genes are governed by different mutation rates, which resulted in different sequence (quasispecies) spaces/sizes in different gene regions.

### Different selection pressures on different domains of E gene of DENV-3

Our previous analysis of the E gene of DENV-3 covered only 131 amino acids [7]. The PCR amplification by primer pair p1259A and cdc2503B in this study covered 1239 nucleotides encoding 413 amino acids, including portion of domain I and II, 3 hinge regions, and complete domain III to the end of the stem-anchor region [12]. However, genetic instability was observed when the PCR product was cloned into the T/A vector and propagated in *E. coli*. The genetic truncation occurred consistently at the location following amino acid position 412 of the envelope gene (E412). This truncation was observed in 29 clones (21.5%) out of 135 clones sequenced. In order to increase the sample size and to investigate the extent of amino acid substitution in the E protein, the deduced amino acid sequences of all 135 clonally obtained sequences from patients' viremic plasma were aligned and trimmed so that it contained 293 amino acids, ranging from E118 to E412, which include portions of domain I and II, the complete domain III and a portion of stem-anchor region for analysis (Table 3). Consistently, there was a higher mean amino acid p-distance in dengue hemorrhagic patients (1H: 0.008 ± 0.002, 2H: 0.012 ± 0.002, 3H: 0.009 ± 0.002) than in dengue fever patients (1F: 0.006 ± 0.001, 2F: 0.007 ± 0.002, 3F: 0.007 ± 0.001) with statistical significance (p < 0.05).

**Table 3:** Mean p-distance and ratio of dN to dS per site of amino acid among different domains of the E protein in DENV-3 infected patients

**Virus ID**	**No of sequences**	**Envelope (293aa)**		**Domain I (70aa)**		**Domain II (106aa)**		**Domain III (100aa)**	
		
		**Mean p-distance**	**dN/dS**	**Mean p-distance**	**dN/dS**	**Mean p-distance**	**dN/dS**	**Mean p-distance**	**dN/dS**
1F	26	0.006 ± 0.001	2.04	0.009 ± 0.003	1.82	0.005 ± 0.002	1.72	0.005 ± 0.002	2.8
1H	21	0.008 ± 0.002	1.11	0.008 ± 0.004	2.53	0.007 ± 0.003	0.59	0.007 ± 0.002	1.2
2F	18	0.007 ± 0.002	1.38	0.009 ± 0.004	2.52	0.006 ± 0.003	1.04	0.007 ± 0.003	2.57
2H	25	0.012 ± 0.002	1.06	0.013 ± 0.004	2.68	0.012 ± 0.004	1.08	0.011 ± 0.003	0.77
3F	23	0.007 ± 0.001	0.81	0.012 ± 0.004	0.96	0.005 ± 0.002	0.45	0.003 ± 0.002	1.76
3H	23	0.009 ± 0.002	1.46	0.014 ± 0.005	4.19	0.006 ± 0.003	0.62	0.005 ± 0.002	120.03
**Average**	22.7	0.008 ± 0.002	1.31	0.011 ± 0.002	2.45	0.007 ± 0.002	0.92	0.006 ± 0.002	21.52

Since the E protein is the major determinant of viral entry, cellular tropism and the target of both humoral and cellular immune selection [13,14], amino acid changes associated with particular site changes were further investigated using Phylogenetic Analysis by Maximum Likelihood program (PMAL) [15]. The non-synonymous (d_N_) to synonymous (d_S_) substitution ratio, referred to as parameter omega (ω) in the model, was calculated with the CODEML program from the PAML package, which analyzed and compared the ω ratios codon-by-codon using the maximum likelihood ratio test among three domains [16]. In this study, the M3 model of codon evolution was used since it often provides the best evidence for positive selection. Although some variations were observed, in general, domains III and I were influenced by positive selection as indicated by the d_N_/d_S _ratio larger than 1, but domain II was influenced by neutral selection, as the d_N_/d_S _ratio was smaller than 1. The average value of d_N_/d_S _was the highest in Domain III (21.52), followed by Domain I (2.45), then Domain II (0.92) (Table 3).

### Phylogenetic analysis of DEN-3 Virus

**Figure 2:**
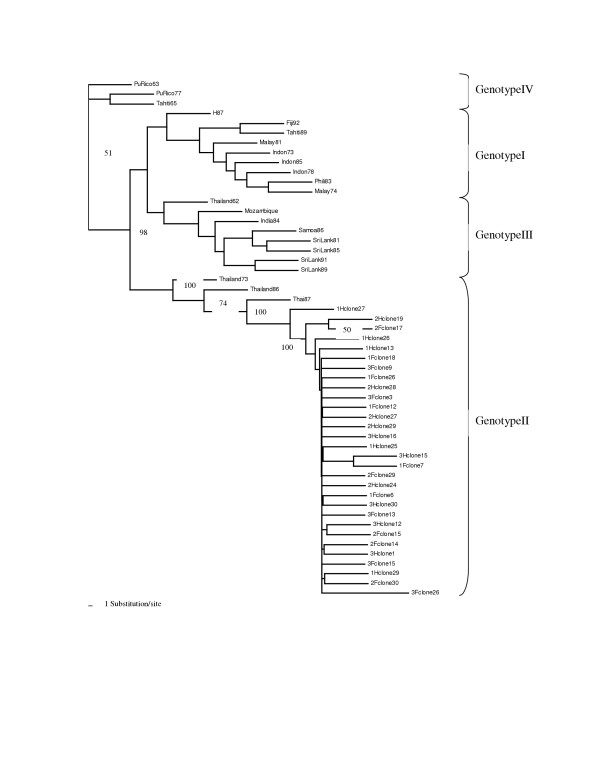
Phylegenetic tree showing the evolutionary relationships of the E gene among 54 sequences from 30 clonal sequences of 6 DEN-3 clinical isolates and 24 global isolates. Bootstrap support values presented as percentage are given for key nodes only and the genotype designations are given. The horizontal branch length of the trees was drawn to scale. GenBank accession numbers of the global DEN-3 strains used in this analysis are as follows: Fiji92 (L11422), India84 (L11424), Indonesia73 (L11425), Indonesia78 (L11426), Indonesia85 (L11428), Malaysia74 (L11429), Malaysia81 (L11427), Mozambique85 (L11430), H87 (L11423), Philippines83 (L11432), Puerto Rico77 (L11434), Puerto Rico63 (L11433), Samoa86 (L11435), SriLanka81 (L11431), Sri Lanka85 (L11436), Sri Lanka89 (L11437), Tahiti65 (L11439), Tahiti89 (L11619), Thailand62 (L11440), Thailand73 (L11620), Thailand87 (L11442).

To determine the evolutionary history of the DENV-3 viruses found in Taiwan in 1998, the nucleotide sequence of their partial E protein genes were compared with those from all previous published DENV-3 E gene sequence available in the GenBank. The phylogenetic tree analysis for 141 clonal sequences from six virus isolates of this study and 24 global DENV-3 sequences separated the viruses into five main subgroups, which had been previously defined as five different genotypes. As in previous studies of DENV-3 diversity, the 1963 Puerto Rico strain formed a distinct outlier, which served as the outgroup for the phylogenetic tree. The tree topology was very similar based on either neighbor-joining (NJ) or parsimony (PAR) method. Based on the phylogenetic tree, the virus isolates from Taiwan in 1998 formed a tight cluster with strong bootstrap support, which fell closer to the isolates from Thailand as they belong to DEN-3 genotype II, according to the classification of Lanciotti et al (20)(Fig 2). Most of the population from the clonal sequences formed a tightly cluster, which represented the highly homogeneous nucleotide sequences during the same epidemic. Interestingly, some clones from different individual isolates appeared to form the different subgroups under the Thailand genotype, with 50–100% bootstrap support. This indicated viral evolution did occur during the epidemic period, probably under selection pressure.

## Discussion and Conclusion

To the best of our knowledge, this was the first systematic attempt to understand the sequence spectrum of the entire genome of DENV-3. Previous studies, focused on certain genomic regions such as the envelope gene, the capsid gene or the NS2B gene, have revealed the presence of quasispecies structure as indicated by the simultaneous presence of multiple variant genomic sequences of the dengue virus isolates from either the clinical samples or field-caught mosquitoes [7,9,17-19]. Instead of arbitrarily choosing one genomic region in this study, the full genomic consensus sequences of six DENV-3 isolates were used to locate the four most prominent heterogeneous regions, the C/PrM and E in the structure, and NS3 and NS5 in the nonstructural regions.

Use of clonal sequencing to study the mutation spectrum needs to ensure that sequencing artifacts due to RT-PCR amplification are reduced to minimum. In this study, we used viral RNAs extracted from patients' viremic plasma directly. In addition, a thermostable polymerase with proof-reading function was incorporated in the RT-PCR, which has been shown to be a simple and valuable method for characterization of mutant spectra of virus quasispecies [20]. The nucleotide changes from four sequenced-viral genomic regions (range of 4.4–11.6 × 10^-5 ^changes/nucleotide/cycle of PCR) were greater than those predicted based on reverse transcriptase (10^-4^) and proof-reading DNA polymerase (*Pfu*, 10^-6 ^error/site/cycle) combined [21]. Based on the experimental data, Arias et al pointed out that the biological and molecular clones were statistically indistinguishable when defining the mutation spectrum with regard to the types and distributions of mutations, mutational hot-spots and mutation frequencies [20]. Similarly, we believe that the full-genomic characterization followed by clonal sequencing procedure employed in this study is a reasonable and justifiable approach for the characterization of mutation spectra (quasispecies dynamic) of DENV-3 viruses.

DENV, like other RNA viruses, exists as quasispecies with the sequence diversity of the envelope gene in the DENV-3 virus population from 6 clinical isolates, ranging from 0.22–0.39% of mean p-distances in this study. These values are within the range calculated by other studies (0.12 to 0.84%) for different portions of the E protein genes of DENV-3 viruses from either the clinical or field-caught mosquito isolates [7,9,17-19]. Our study confirmed use of the structural protein, especially the E gene with higher sequence heterogeneity to study the viral quasispecies, instead of NS protein and 5' and 3' NCR. The extent of sequence variation observed in this study was similar to or lower than what has been reported for acute infection of HIV-1 or HCV [8,22-25]. A study of sequence variation of HIV-1 after sexual transmission revealed that the nucleotide mean diversity of the E gene (gp120) was 0.24% and that of the gag gene (p17) was 0.5% [22]. Similar results by studying variants of hepatitis C virus (HCV) from a single infected blood donor and 13 viraemic recipients were traced to examine the sequence diversity in hypervariable region 1 with sequence p-distance ranged from 0.3% to 6.2% [23]. These data might support an important concept in the evolution of arthropod-borne RNA viruses (arboviruses) which evolve more slowly than RNA viruses transmitted by other routes due to intrinsic constraints associated with dual replication in mammalian and invertebrate hosts [26]. Consistent with this interpretation was that the lower sequence diversity was observed at the same E protein gene from the field-caught mosquito DENV-3 isolates [19] or after inoculation of clinical serum of DENV-3 into mosquitoes (data not shown).

The larger mutation spectra in structural proteins than non-structural proteins probably imply less genetic constraint on the structure proteins to maintain proper function than non-structural proteins. However, the mutations in the structural or non-structural proteins did not accumulate randomly during replication. The mutation rates vary in different functional/structural domains. Even within the envelope structure protein, where domain III, the proposed receptor-binding and neutralizing antibody-binding sites [13] had highest sequence heterogeneity than Domain I or II. The detail analysis in our study further indicated that the different selection pressure was exerted on different domain of the E gene of DENV-3. Domain III and domain I were under the influence of positive selection (d_N_/d_S_:21.52, 2.45) and domain II was under the influence of neutral selection (d_N_/d_S_:0.92). The particularly higher d_N_/d_S _ratio in domain III of viral isolates 3H was caused by the value of 0 of d_S _at the denominator. The positive selection on the domain III is not surprising since domain III contains the receptor-binding domain and major type-specific neutralization epitopes [12]. However, the complete E gene sequence may be required to clarify the evolutionary selection on domain I and II due to incomplete sequence obtained in this study.

In contrast to other studies which suggested the strong purifying selection in the E gene of dengue virus evolution, the consensus sequences used for analysis represented dengue viral gene conservation during long-term evolution [27]. The clonal sequences obtained from our study represented the selection pressure imposed on viral populations during the short term of evolution, which might explain the substantially different d_N_/d_S _value within hosts and among genotypes. The majority of the nonsynonymous mutations that arise within each host occurred as singletons with relatively low frequency in the population; thus are likely to be deleterious. Such heterogeneous gene pool may give rise to various viruses able to occupy new ecological niches or to adapt to sudden selection pressures on the cycle of replication. It is evident that certain nonsynonymous nucleotide mutations at specific sites repeatedly occurred among different virus isolates as well as after mosquito inoculation in our study (data not shown), which has been proposed as quasispecies memory in another study [28]. Further studies are needed to address the influence of these mutations on the overall fitness in different hosts (i.e., mosquito and human) during dengue viral transmission.

## Materials and methods

### Study subjects and virus isolation

Six dengue patients were identified by RT-PCR to be DENV-3 positive during the 1998 epidemic and their acute-phase viremic plasma samples were collected within seven days following the onset of fever. These plasma samples were used to infect C6/36 *Aedes albopictus *mosquito cell lines as described previously [29]. The study protocol was approved by the College of Public Health Research Ethics Review Committee at the National Taiwan University with the informed consent obtained from six dengue patients. Six adult dengue cases between 38 and 63 years of age, including one DF (F) and one DHF (H) cases, whose disease status were classified based on WHO criteria [30], were represented as 1F, 1H, 2F, 2H, 3F and 3H, respectively.

DENV-3 was confirmed by indirect immuno-fluorescent antibody (IFA) tests using serotype-specific monoclonal antibodies (DENV-1:H47, DENV-2:H46, DENV-3:H49, DENV-4:H48) [31]. The C6/36-passage one viral stock was used for full genomic consensus sequencing to identify regions with sequence heterogeneity for clonal sequencing as described later.

### Preparation of viral RNA, RT-PCR amplification and consensus sequencing of PCR products

Viral RNA was extracted either from viremic plasma specimens or from the C6/36-passaged one cell culture fluids using QIAamp viral RNA mini kit (Qiagen, Germany) by following the manufacturer's protocol. The eluted RNA was used as the template and overlapping regions of DENV-3 genome amplified by Titan™ one tube RT-PCR System (Boehringer Mannheim, Germany) following the manufacturer's suggestions. The oligonucleotide primer pairs were designed based on published full-length DENV-3 sequence data for the strains of H87 and 80-2 (GenBank Accession number M93130 and AF317645) and the unpublished DENV-3 sequences (Chang, G-J. personal communication). Ten overlapping fragments were generated which spanned genomic regions of DENV-3 at the following nucleotide (nt) positions: 1 to 1199, 540 to 1712, 1259 to 2521, 2171 to 3435, 3142 to 4697, 4124 to 5708, 5443 to 7497, 7246 to 8770, 8501 to 10335, 9991 to 10709. Primer sequences used for PCR amplification were summarized in Table 4. The obtained PCR products were sequenced by using the Big Dye Terminator Sequencing kit (Perkin-Elmer, Applied Biosystems, Foster City, CA) and analyzed by the 3100 automate sequencer (Perkin-Elmer, Applied Biosystems) with a short capillary.

### Preparation of plasmid templates for clonal sequencing

We used pCRII-TOPO™ T/A cloning kit (Invitrogen, San Diego, CA) to clone PCR products representing heterogeneity sequence regions identified by the consensus sequencing protocol at the previous section. The T/A vector ligated PCR product was used to transform *Escherichia coli *TOP10 competent cells (Invitrogen) and at least 30 white colonies were picked, to grow in 3 ml LB broth at 37°C overnight. Plasmid DNAs were extracted by the QIAprep Spin Miniprep kit (Qiagen), and each plasmid DNA with the desired inserts was completely sequenced using insert flanking primers, T7 and cSP6.

### Nucleotide and Amino acid sequence analysis

Overlapping chromatogram files retrieved from the automate sequencer were analyzed and edited using the SeqMan program in the Lasergene software package (DNASTAR inc., Madison, WI). The derived consensus sequences after excluding the sequences of amplifying primers were aligned using GCG package (Genetic Computer Group, WI). For full-length genomic sequences we paid special attention to identify the regions which consistently presented mixed-chromatographic peaks in the trace file obtained from at least two independent sequencing primers. These regions were selected for the follow-up clonal sequence analysis. Pairwise comparisons of both nucleotide and amino acid sequences between isolates and clonal sequences were performed using the program MEGA v3.0 (Molecular Evolutionary Genetics Analysis, Pennsylvania State University, PA) to determine the numbers of transition and transversion changes, and the mean and proportion of difference, Hamming distance and p-distance, as described previously [8,32,33]. Synonymous (d_S_) and nonsynonymous (d_N_) distances relative to the consensus sequences were calculated within each isolate by maximum likelihood ratio method in the CODEML program from the PAML package [15]. Instead of assuming that all sites are under the same selection pressure with the same underlying d_N_/d_S _ratio, it allows variable selection intensity to vary among amino acid sites [34,35]. In this study, M3 model of codon evolution was applied for which often provides the best evidence for positive selection [16]. An excess of nonsynonymous substitutions over synonymous substitutions (ie. the ratio of d_N_/d_S _> 1) is an indicator of positive natural selection at the molecular level.

The results were expressed as the mean ± standard deviation (SD). T-tests were performed on two-sampled tests and a one-way ANOVA was performed to compare data from different genomic regions, family clusters or different domains in envelope region. In all tests, a p-value less than 0.05 was considered statistically significant.

### Evolutionary analysis

The nucleotide sequences generated in this study were combined with those of all other DENV-3 E protein gene sequences available on GenBank, which resulted in a total data set of 154 sequences. Phylogenetic trees were estimated using parsimony method available in the Phylip v3.6 package [36]. Bootstrap resampling analysis of 500 replicates was generated with the SEQBOOT program to prove the stability of the trees. Phylogenetic trees were delineated using the TreeView (v.1.6.6) program by using Puerto Rico 1963 isolate as the outgroup. For better presentation of the phylogenetic tree, only 30 clonal sequences from six different clinical isolates and 24 global isolates were shown in Fig 2.

### Nucleotide sequences accession numbers

The sequences from four heterogeneous regions of dengue viruses from the six patients studied here have all been submitted to GenBank, and their accession numbers are from DQ109039 to DQ109173 for the E region, from DQ109174 to DQ109305 for the capsid/prM region, from DQ109306 to DQ109405 for the NS5 region and from DQ109406 to DQ109524 for the NS3 region.

## Competing interests

The author(s) declare that they have no competing interests.

## Authors' contributions

DYC designed and performed all the experiments and helped drafted this manuscript. CCK helped with collecting field isolates and instructed the experiments, together with WKW and HLW. WJC helped for the mosquito injection experiments and GJC formulated the idea for this study and also provided critical comments regarding this manuscript.

## Financial support

The study was supported by the grants from the National Health Research Institute (NHRI), Taipei, Taiwan (grant number: NHRI#DD01-861X-CR-501P and NHRI#CN-CL8903P) and International Society of Infectious Disease (ISID).
